# Photonic Readout of Superconducting Nanowire Single Photon Counting Detectors

**DOI:** 10.1038/s41598-020-65971-5

**Published:** 2020-06-11

**Authors:** Marc de Cea, Emma E. Wollman, Amir H. Atabaki, Dodd J. Gray, Matthew D. Shaw, Rajeev J. Ram

**Affiliations:** 1 Research Laboratory of Electronics, Massachusetts Institute of Technology, Cambridge, MA 02139 USA; 2 Jet Propulsion Laboratory, California Institute of Technology, Pasadena, CA 91109 USA

**Keywords:** Superconducting devices, Fibre optics and optical communications, Integrated optics, Optoelectronic devices and components

## Abstract

Scalable, low power, high speed data transfer between cryogenic (0.1–4 K) and room temperature environments is essential for the realization of practical, large-scale systems based on superconducting technologies. A promising approach to overcome the limitations of conventional wire-based readout is the use of optical fiber communication. Optical fiber presents a 100–1,000x lower heat load than conventional electrical wiring, relaxing the requirements for thermal anchoring, and is also immune to electromagnetic interference, which allows routing of sensitive signals with improved robustness to noise and crosstalk. Most importantly, optical fibers allow for very high bandwidth densities (in the Tbps/mm^2^ range) by carrying multiple signals through the same physical fiber (Wavelength Division Multiplexing, WDM). Here, we demonstrate for the first time optical readout of a superconducting nanowire single-photon detector (SNSPD) directly coupled to a CMOS photonic modulator, without the need for an interfacing device. By operating the modulator in the forward bias regime at a temperature of 3.6 K, we achieve very high modulation efficiency (1,000–10,000 pm/V) and a low input impedance of 500 Ω with a low power dissipation of 40 *μ*W. This allows us to obtain optical modulation with the low, millivolt-level signal generated by the SNSPD.

## Introduction

While promising, optical readout of cryogenic devices is challenging. First, we need semiconductor electro-optic devices operating at cryogenic temperatures, where effects such as carrier freeze-out (the incomplete ionization of p- and n-type dopants due to reduced thermal energy) can hinder device performance^1^. Second, while superconducting devices have intrinsically low resistance, typical input impedances for electro-optic modulators are high (>100 *k*Ω). This impedance mismatch makes direct delivery of electrical signals from the superconducting device to the modulator challenging. Third, we need to operate with the mV-range electrical signals characteristic of superconducting electronics, while driving signals for conventional room temperature electro-optic modulators are in the 0.5 V–2 V range.

To overcome these limitations, previous demonstrations have relied on the use of an interfacing device between the superconducting and the electro-optic devices. The use of semiconductor amplifiers is possible^2–7^, but its mW-scale power dissipation hinders its scalability. Another alternative is to use a nanocryotron^8^, but this requires actively resetting the device every time a pulse is generated. Recently, the use of a cryogenic thermal switch to drive a laser diode with low power dissipation has been reported^9^, but a slow turn-off time of 15 ns limits the achievable bandwidth of this approach.

**Figure 1:**
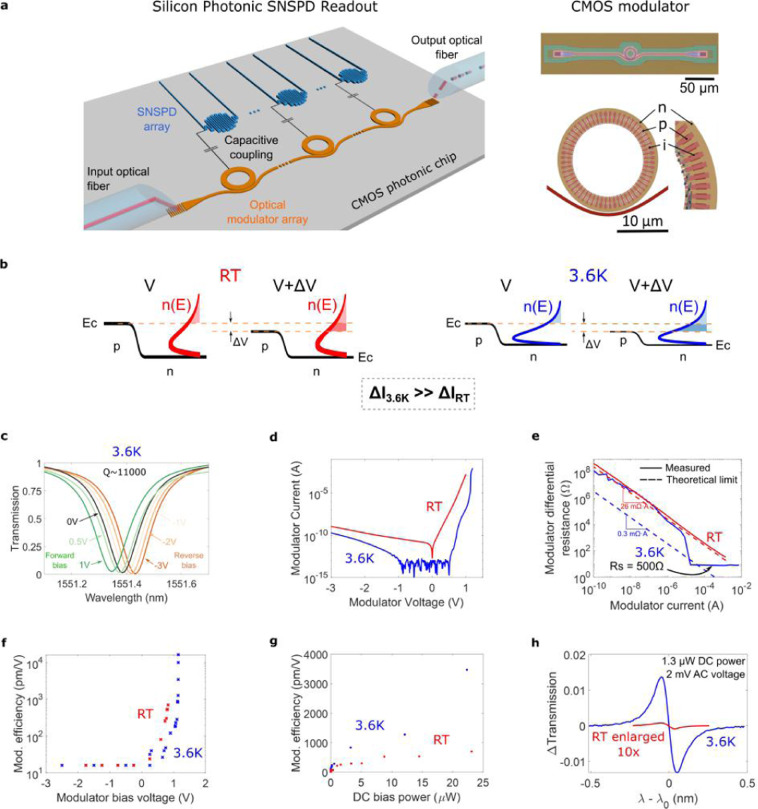
Forward biased CMOS modulator for cryogenic optical readout. (**a**) Optical readout system. The superconducting device (an SNSPD here) directly drives an optical modulator, which encodes the data into an optical carrier. Right: Micrograph (top) and layout (bottom) of the T-shaped silicon ring modulator. (**b**) Modulator’s p-n junction conduction band and free electron distribution $n(E)$ for voltages V and V + Δ*V*. Due to $n(E)$ being tightly distributed at low temperatures, the same Δ*V* results in a stronger current injection. (**c**) Modulator’s transmission spectra at different bias voltages. (**d**) Modulator’s I-V curve. Low temperature operation increases the turn-on voltage (due to increased built-in potential) and the I-V slope (because of tighter $n(E)$ distribution). (**e**) Modulator’s differential resistance (${r}_{d}=dV/dI={k}_{B}T/qI+{R}_{s}$). At 3.6 K and currents >5 *μ*A, ionization decreases the series resistance. (**f**) Modulation efficiency versus voltage. An exponential increase is measured in forward bias. (**g**) Modulation efficiency versus DC electrical power. Higher efficiency is obtained for the same power at 3.6 K. (**h**) Transmission change versus detuning between laser wavelength *λ* and resonance wavelength *λ*_0_ for a 1.3 *μ*W DC power consumption and 2 mV AC signal. Increased modulation efficiency makes Δ*T* much stronger at low temperatures.

Here, we use a silicon optical modulator biased in the forward regime. Because of its high efficiency, modulation of the optical carrier is achieved with the small voltages generated by the SNSPD. Because of its low input impedance, direct delivery of the SNSPD signal to the modulator is possible. Therefore, we realize optical readout without the need of an interfacing device (Fig. 1(a)).

Optical communication is becoming the preferred I/O solution in modern, room temperature high performance systems^10,11^, and this work demonstrates its suitability for scalable cryogenic readout. This work could help achieve the full potential of superconducting technologies, with applications in a broad range of fields including quantum computing^12,13^, superconducting electronics^14,15^, single photon imaging^16^ and space-based communications^17,18^.

## Results

### Forward bias operation of silicon modulators at 3.6 K

#### CMOS photonic resonant modulator

Our optical modulator is a ‘spoked’ silicon microring resonator (Fig. 1(a)) with p–n junctions interleaved along the azimuthal dimension^19^, fabricated using a commercial high-performance 45 nm CMOS silicon-on-insulator (SOI) process (see Methods). The ring exhibits a sharp, notch-filter optical transmission with a stop-band at the resonant wavelength of the ring ${\lambda }_{0}$. Applying a voltage across the junctions modulates the free carrier concentration (electrons and holes), which influences the refractive index of the ring waveguide due to the plasma dispersion effect^20^ and shifts ${\lambda }_{0}$^21^ (Fig. 1(c)). By modulating ${\lambda }_{0}$, the transmission of a continuous wave optical carrier can be modified, achieving optical modulation^22,23^. Arrays of devices with varying microring diameters can be used to implement WDM transmitters^24,25^.

#### Forward bias operation: experimental results and discussion

To achieve modulation with mV signals, we operate the modulator in forward bias ($V > {V}_{ON}$). In this regime, the change in carrier density is due to carrier injection, which depends exponentially on voltage^26^. This is in contrast with reverse bias operation ($V < 0$), where the carrier density change comes from modifying the depletion region width of the p-n junction, with a much weaker voltage dependence ($\sqrt{V}$) and increased sensitivity to doping density (and hence carrier freeze-out). In reverse bias the modulation efficiency - the change in resonance wavelength with applied voltage - ranges from the 16 pm/V we measured for our device to 250 pm/V for the highest performing device reported in the literature^23^. In forward bias and at 3.6 K, we measured modulation efficiencies reaching 1000 pm/V at 7 *μ*A bias and 10,000 pm/V at 40 *μ*A (Fig. 1(f)).

Despite significantly higher modulation efficiency, forward bias is rarely used because of increased power consumption (due to static current flowing through the p-n junctions) and lower modulation speed. The injected charges are not removed by a strong electric field (as happens in reverse bias) but must recombine to reset the device state, which happens on the scale of the carrier recombination lifetime (~ns). The maximum measured bandwidth for our device at room temperature is 9 GHz in reverse bias, but only 1 GHz in forward bias (see Supplementary Data 1). Likewise, the static power dissipation in reverse bias is only 200–500 pW whereas in forward bias it is 0.1–100 *μ*W.

Cryogenic operation fundamentally changes the performance trade-off between forward and reverse bias. At cryogenic temperatures the carriers are distributed over a narrow range of energy states within the conduction and valence bands. Thus, as shown in Fig. 1(b), a stronger change in the number of injected carriers for the same differential voltage Δ*V* is obtained compared to room temperature, resulting in a larger change in current as seen in the increased IV curve slope (Fig. 1(d)). Therefore, at cryogenic temperatures the same differential carrier injection (and thus the same modulation efficiency) is achieved at a lower bias current (and thus at lower static electrical power consumption). This is shown in Fig. 1(g): to achieve a modulation efficiency of 700 pm/V, 23 *μ*W of DC electrical power are needed at room temperature, compared to only 1.1 *μ*W at 3.6 K. Figure 1(h) further illustrates this: for a 2 mVpp driving signal and 1.3 *μ*W DC electrical power dissipation, less than 0.01% change in transmission is achieved at room temperature, whereas at 3.6 K we can achieve a transmission change larger than 1%.

Electron-hole recombination lifetimes exhibit small changes as temperature drops – bimolecular recombination increases whereas Shockley-Read-Hall (defect assisted) recombination may decrease slightly. As a result, bandwidth in forward bias is relatively independent of temperature: 0.9 GHz at 300 K versus 1.5 GHz at 4 K (see Supplementary Data 1). On the contrary, in reverse bias the bandwidth is limited by the resistance and capacitance (RC time-constant) of the p-n junctions, which are highly dependent on the number of ionized dopants and therefore on temperature. As carrier freeze-out occurs, bandwidth decreases precipitously: 9 GHz at 300 K versus 0.2 GHz at 4 K (see Supplementary Data 1). While this can be mitigated by increasing doping densities^27^, this comes at a cost of increased optical loss, reduced resonator quality (Q) factor and decreased modulation efficiency.

Because of the rectifying property of p-n junctions, a modulator under reverse bias presents a purely capacitive input impedance, but forward bias adds a resistive component, lowering the input impedance and reducing the mismatch between the superconducting device and the modulator. As temperature decreases, dynamic resistance (${r}_{d}={R}_{s}+kT/qI$, where ${R}_{s}$ is series resistance, *k* is the Boltzmann constant and *I* is the current through the p-n junction^26^) drops, further reducing the impedance mismatch. ${R}_{s}$ is sensitive to carrier freeze-out, but the small current flowing through the forward-biased diode can ionize the dopant atoms, maintaining a low ${R}_{s}$. We measure a forward-biased impedance that reduces from 2 *k*Ω at 300 K to 500 Ω at 3.6 K – limited by ${R}_{s}$ (Fig. 1(e)). As we demonstrate, superconducting electronics can drive this input impedance.

### Photonic readout of an SNSPD

#### Operating principle

An SNSPD consists of a narrow wire patterned from a thin superconducting film that behaves as a switch that is activated by the detection of a single photon. When a photon is absorbed, the superconducting film develops a local resistive region, or hotspot, with a resistance on the order of kΩ. By detecting this resistance change through a readout circuit, a single photon detector can be realized. We used a Molybdenum Silicide (MoSi) SNSPD optimized for UV photon detection^28^ (see Methods).

**Figure 2:**
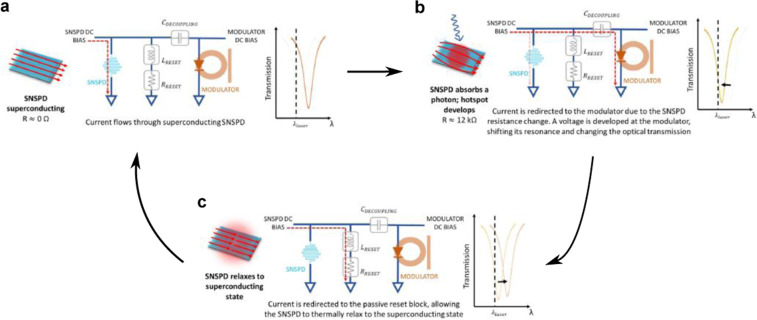
Working principle of the SNSPD optical readout. (**a**) The superconducting SNSPD provides a low impedance path to ground so all the bias current flows through it. (**b**) When the SNSPD absorbs a photon, the developed hotspot resistance diverts the current into the readout, producing a voltage pulse that drives the modulator and shifts its resonance, therefore changing the transmitted light. (**c**) The passive reset circuit provides a low-impedance path to ground, allowing for the hotspot to thermally relax and for the SNSPD to go back to its superconducting state. *C*_DECOUPLING_ = 100 pF, *L*_RESET_ = 8 *μ*H, *R*_RESET_ = 50 Ω.

Figure 2 shows the optical readout circuit and its operation. A decoupling capacitor (${C}_{{\rm{DECOUPLING}}}=100$ pF) is added to allow for separate DC biases to each device while coupling the AC signal generated by the SNSPD into the modulator. When the SNSPD is superconducting, it provides a low impedance path to ground so all the bias current flows through it (Fig. 2(a)). After the SNSPD absorbs a photon, the developed hotspot resistance (~12 kΩ) diverts most of the current into the readout, producing a voltage pulse that drives the modulator and shifts its resonance, changing the intensity of the transmitted light (Fig. 2(b)). A reset circuit (${L}_{{\rm{RESET}}}=8$ *μ*H, ${R}_{{\rm{RESET}}}=50$ Ω) provides a low-impedance path to ground, diverting any leftover current from the nanowire, allowing for the hotspot to thermally relax and for the SNSPD to return to its superconducting state (Fig. 2(c)).

**Figure 3:**
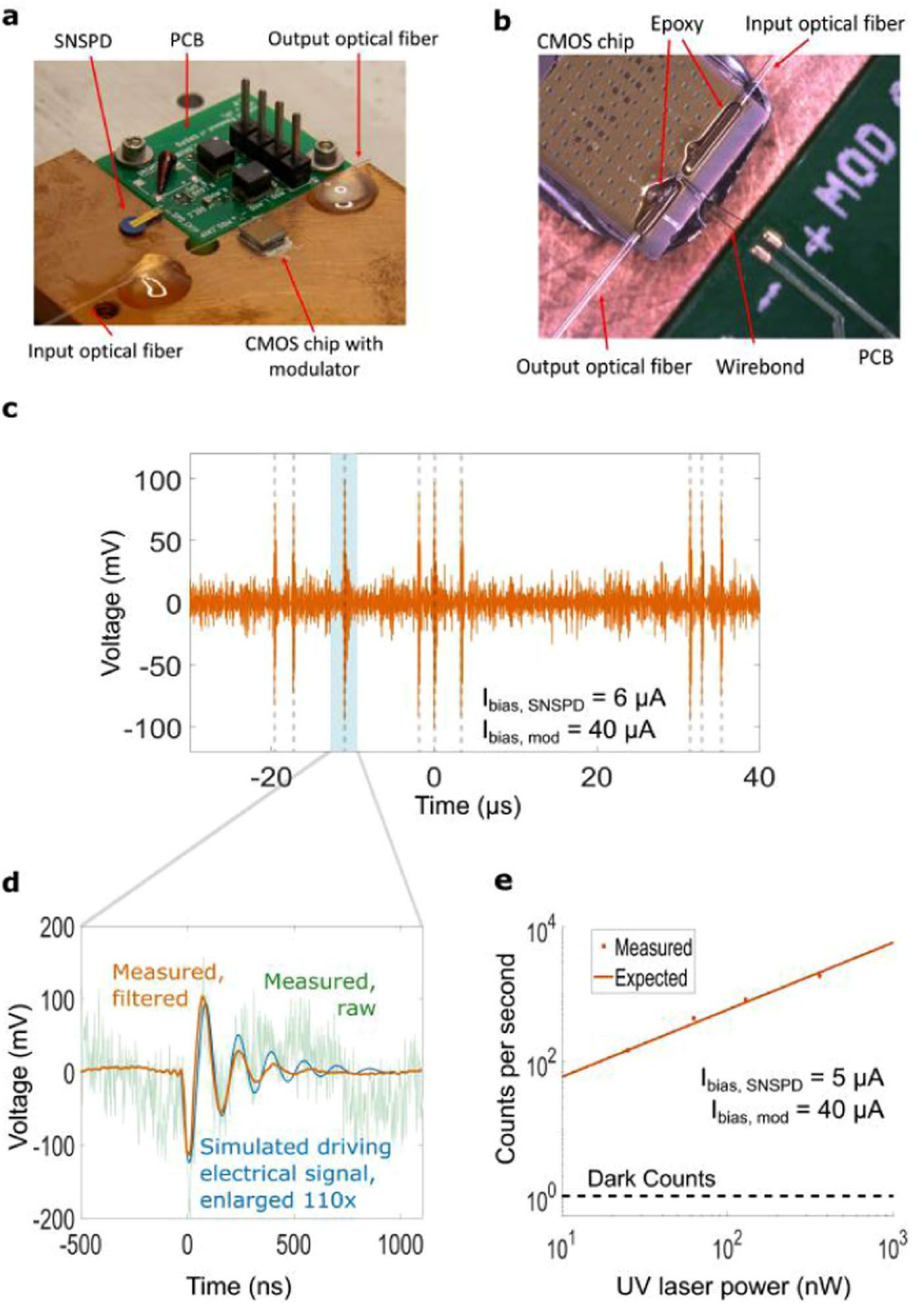
Optical readout of an SNSPD. (**a**) Picture of the assembly. (**b**) Fiber attach. Input and output optical fibers are aligned to vertical grating couplers and epoxied to the CMOS chip. (**c**) Filtered optical readout signal. SNSPD triggering events are highlighted. (**d**) Readout pulse generated by a single photon. Orange shows a filtered signal (see Supplementary Methods 2), light green a single readout pulse and blue depicts the simulated electrical signal driving. (**e**) Measured (dots) and expected (solid line) counts registered by the optical readout as a function of UV power incident on the SNSPD.

Figure 3(a) shows the packaged readout system. The SNSPD and modulator chips are wirebonded (Fig. 3(b)) to a circuit board which implements all the passive components necessary for the readout. The assembly was mounted on the 3.6 K stage of the cryostat, and the output optical fiber was connected to a room temperature high speed photodetector (see Methods).

#### Experimental characterization

Figure 3(c) shows a typical readout waveform recorded using a high speed oscilloscope. Each pulse represents a single photon incident on the SNSPD that is imprinted on the intensity of the readout optical signal. 1 mW of input 1550 nm light was used for readout of the modulator, corresponding to 30 *μ*W on chip after the input grating coupler (with 15 dB loss, see Methods). The SNSPD bias was 6 *μ*A, while the modulator was biased at 40 *μ*A, corresponding to a modulation efficiency of 10,000 pm/V, a 45 *μ*W electrical power dissipation and an input resistance of 500 Ω. Figure 3(d) shows the readout pulse generated by a single photon detection event. The signal differs from typical SNSPD pulses and shows slowly decaying oscillations due mainly to the parasitic capacitance introduced by the SNSPD chip, which is not optimized to minimize stray capacitance. The peak to peak amplitude of the driving electrical signal (obtained by simulation, see Supplementary Discussion 1) is only 2 mV: because of forward bias operation, modulation is achieved with this small signal, which would not be enough in reverse bias. With such a small amplitude, the AC electrical power is at least two orders of magnitude lower than the DC power consumption (see Supplementary Discussion 2). Thus, the latter dominates the total electrical power dissipation in our readout. With a measured bandwidth of 1.5 GHz, the modulator is fast enough to respond to the SNSPD signal, and is faster than the 500 MHz bandwidth electrical amplifiers typically used in SNSPD readout^29,30^. We measured the number of counts for different UV powers incident on the SNSPD showing that, as expected, the readout behaves linearly with incident power (Fig. 3(e)).

## Discussion

While operation of a reverse-biased silicon ring modulator at 4.2 K has been previously reported^27^ and a cryogenic modulator based on the Pockels effect in BaTiO_3_ has been recently presented^31^, this work constitutes, to the best of our knowledge, the first demonstration of readout of a superconducting device through an optical modulator, and a demonstration of the low input impedance and high modulation efficiency achievable in forward biased silicon modulators at cryogenic temperatures.

With 40 *μ*W electrical power dissipation, our optical readout presents 100x lower heat load than typical readout schemes using a cryogenic amplifier, and is 10x faster than the thermal switch reported by Mccaughan *et al*.^9^. While our demonstration was limited by high optical coupling losses, simple improvements would result in a readout limited only by the internal efficiency of the SNSPD and could reduce the necessary optical power from the 1 mW used in this work to 5 *μ*W (see Supplementary Discussion 4). This demonstration opens up the path to the realization of scalable, low power, high throughput communication between cryogenic and room temperature environments, addressing one of the key remaining challenges for the wide adoption of cryogenic technologies.

## Methods

### Optical Modulator

The silicon microring modulator used in this work is designed to work at a wavelength of 1550 nm, has an outer radius of 10 *μ*m, is 1.7 *μ*m wide and roughly 100 nm thick^32^. The chip was fabricated using a commercial high-performance 45 nm complementary metal–oxide semiconductor (CMOS) silicon-on-insulator (SOI) process, without any modification to the process flow, in what is known as zero-change CMOS^33^. The ring is realized in the crystalline-silicon layer, and the standard CMOS implants aimed for transistor fabrication are used to implement the different doping regions that form the interleaved p-n junctions, complying with all the foundry design rules. This provides a low cost, highly scalable photonic platform that can be monolythically integrated with electronics^11^. By exploiting the maturity of this platform, the possibility of building large arrays of these modulator devices for high throughput readout of large cryogenic systems in a cost effective way is readily accessible.

### SNSPD

The MoSi detector has a 10 nm × 110 nm cross-section, a 180 nm pitch with a 56 *μ*m diameter active area and shows a detection efficiency of about 70% at 373 nm^28^ (Supplementary Methods 1). This device has a high inductance of ~12.8 *μ*H, allowing it to develop a large hotspot resistance during photodetection events. Fits to the rising edge of a typical pulse from the device give a hotspot resistance of approximately 12 kΩ. Compared to typical near-IR SNSPDs with smaller active-areas, this high impedance allows the detector to drive a larger load resistance and therefore produce a larger voltage signal at the modulator. The UV detector also has over 60 dB of rejection at 1550 nm, making it less sensitive to any scattering of the light used for the readout. The detector has a higher operating temperature compared to typical near-IR SNSPDs, with a switching current above 10 *μ*A at temperatures below 3.8 K.

### Printed Circuit Board

A PCB was designed to interface the SNSPD chip with the modulator. The bottom layer was completely gold plated to maximize thermal contact to the cold head and ensure correct thermalization. The maximum available FR-4 dielectric thickness of 3.2 mm was used to minimize the parasitic capacitance. Air-coil inductors, silicon capacitors and thin film resistors were used to ensure performance at cryogenic temperatures. Bond pads were included to allow connection of the SNSPD and modulator chips through wirebonds. Aluminum was used for the SNSPD, whereas gold wirebonds were used for the modulator. While in this work we used a PCB, it is possible to integrate all the passive components in the CMOS chip to allow for direct interfacing between the modulator and SNSPD chips.

### Cryogenic Fiber Attach

One of the most challenging aspects of fiber based cryogenic optical readout is the need for a reliable, robust and repeatable fiber attach method capable of surviving the thermal stresses associated to the cooling from room temperature. Our CMOS chip uses vertical grating couplers designed for a 5 *μ*m mode field diameter (MFD) to couple light into and out of the chip. These structures have stringent misalignment tolerances of about 1 *μ*m. Thus, the fiber attach mechanism has to maintain the fiber tip position within 1 *μ*m throughout the whole process of placing the system into the cryostat and cooling it down to 3.6 K.

A similar approach to the one described by McKenna *et al*.^34^ was used. Angle-polished fibers matched to the design angle of the grating couplers (≈15 degrees with respect to the direction normal to the chip) were glued to the chip after optimization of the alignment with micropositioners using Norland Optical Adhesive 88 (NOA 88). A 365 nm UV LED was used to cure the NOA, and the attach was left sitting at RT for 24 hours to ensure optimal adhesion. Two different gluing steps were performed. First, a small amount of NOA was deposited and cured at the fiber tip to ensure it is correctly held in place. Second, a large amount of NOA was deposited away from the tip to serve as stress relief and ensure that any movement of the rest of the fiber does not affect the highly sensitive fiber tip attach.

Optimal alignment of SMF-28 fibers with a 10 *μ*m MFD to the grating couplers in the CMOS chip results in 10 dB insertion loss per grating coupler. Due to tolerances in the polish angle and non-perfect alignment, we incurred in around 3.5 dB of extra loss after curing of the epoxy at room temperature. During the cooldown from RT to 3.6 K, 1.5 dB was lost due to thermal contraction. These result in a total loss of about 15 dB per grating coupler, which translates into a 30 dB total insertion loss between the input and output of the cryostat.

These high losses are not intrinsic to the technology. Grating couplers with >90% efficiency have been demonstrated in our CMOS photonic platform^35^. With the use of optimized grating couplers and a better polish angle control, total insertion losses could be reduced to about 3–5 dB after cooling down to cryogenic temperatures.

### Experimental Setup

The SNSPD and modulator were both operated on the 2nd stage of a two-stage Gifford-McMahon (GM) cryocooler. The output optical signal from the cryostat was connected to a high speed photodetector (New Focus 1544B), and the resulting electrical signal was amplified using a Low Noise Amplifier (Mini Circuits ZKL 1R5+). A low pass filter was then used to filter out high frequency noise, and its output connected either to a high speed oscilloscope (Agilent DSO81204A) or a pulse counter (Agilent 53131A). To overcome the high optical insertion loss of 30 dB coming from the non optimized grating couplers, an EDFA (JDSU Erfa 1215) was used before the cryostat input to amplify the light coming from a C band tunable laser (New Focus TLB-6600), and a variable optical attenuator (Ando AQ8201-31) was used to control the optical power getting into the cryostat. For the same reason, an additional EDFA followed by a narrowband filter (Agiltron FOTF) to filter out ASE noise was used at the output of the cryostat before going into the photodetector. A UV laser (PicoQuant LDH-P-C-375) followed by a chain of optical filters was used to control the amount of UV light hitting the SNSPD.

## Supplementary information

Supplementary Information.
