# Supplementary material for: Photonic Readout of Superconducting Nanowire Single Photon Counting Detectors
Source: Sci Rep. 2020 Jun 11;10:9470. doi: 10.1038/s41598-020-65971-5 (PMC7289839; doi:10.1038/s41598-020-65971-5)
Supplement: Supplementary file 1 — Supplementary Information. [file 41598_2020_65971_MOESM1_ESM.pdf]

Supplementary Material - Photonic Readout of Superconducting Nanowire Photon Counting Detectors

Marc de Cea[1], Emma E. Wollman[2], Amir H. Atabaki[1], Dodd J. Gray[1], Matthew D. Shaw[2], and Rajeev J. Ram[1,*]

[1]Research Laboratory of Electronics, Massachusetts Institute of Technology, Cambridge, MA 02139, USA.
[2]Jet Propulsion Laboratory, California Institute of Technology, Pasadena, CA 91109, USA.
[*]rajeev@mit.edu

Supplementary Data 1: CMOS modulator characterization

Fig. 1(f) in the main text shows the modulation efficiency as a function of voltage both at room temperature and 3.6K. While this is the preferred way to report modulation efficiency in the literature, in the forward bias regime it is informative to plot the modulation efficiency as a function of bias current. This is shown in Fig. 1.

We also characterized the bandwidth of our modulator in the forward and reverse bias regime, both at room temperature and 4K. The results are shown in Fig. 2.

As discussed in the main text, the 3 dB bandwidth in reverse bias at room temperature is high and close to 9 GHz. Nevertheless, it drops to about 200 MHz at 4K because of the increased resistance of the p-n junction quasi-neutral regions caused by the freezeout of the carriers. This generates an increase in the RC time constant of the device, which limits its bandwidth. These results are in good agreement with previously reported results[1].

In forward bias, the 3 dB bandwidth is close to 900 MHz at room temperature, and it increases to 1.5 GHz at 4K. As discussed in the main text, the bandwidth limit in the forward bias regime is set by the minority carrier lifetime in the ring waveguide, which does exhibit relatively low dependence with temperature. Our measurement results suggest that the carrier lifetime decreases slightly at low temperatures, which is explained by an increase in both radiative and Shockley-Read-Hall recombination[2,3].

Supplementary Methods 1: SNSPD

A micrograph of the Molybdenum Silicide (MoSi) SNSPD used in this work is shown in Fig. 3(a), and its cross section depicted in Fig. 3(b). The detector is optimized for UV light, and has an internal quantum efficiency of 70% at 365 nm (Fig. 3(c)). A detailed characterization of this device is presented elsewhere[4].

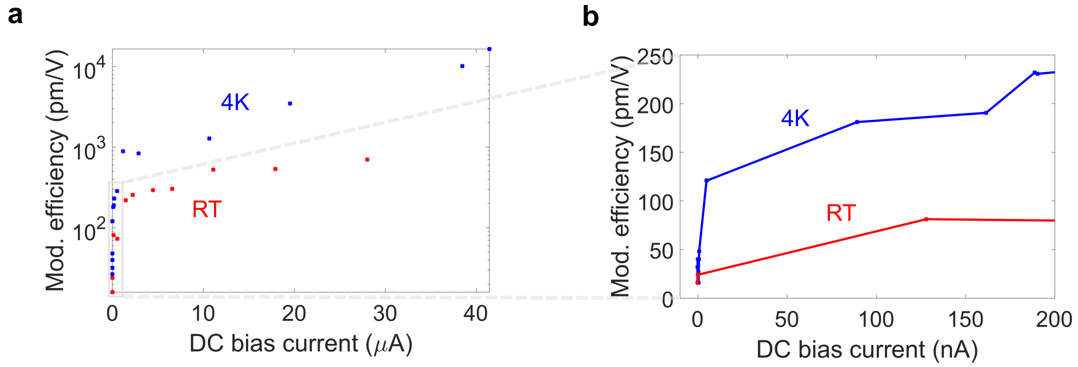

Figure 1. Modulation efficiency as a function of bias current. A closeup for reverse bias and weak forward bias currents is shown in (b).

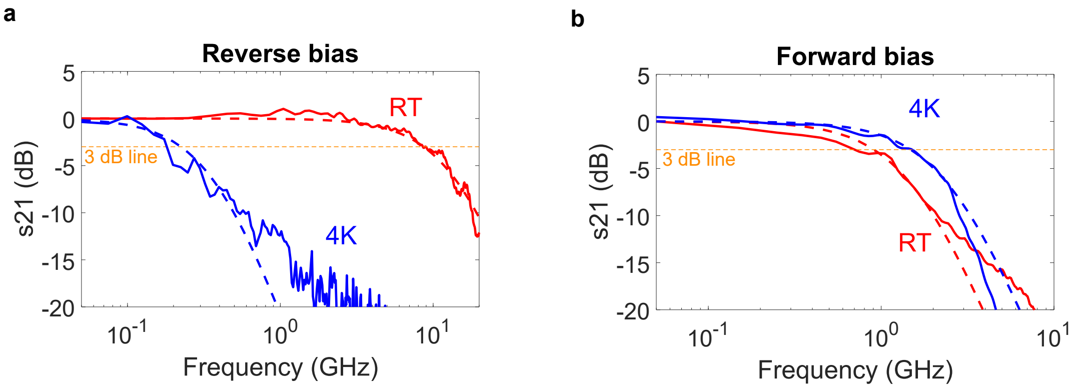

Figure 2. Modulator bandwidth. (a) Bandwidth for -3 V reverse bias. (b) Bandwidth for a 20 μA forward bias. This corresponds to a bias voltage of 0.8V at RT and 1.13V at 4K. For both (a) and (b), red depicts the room temperature results and blue corresponds to 4K. Dashed lines show the best fit to a single pole transfer function.

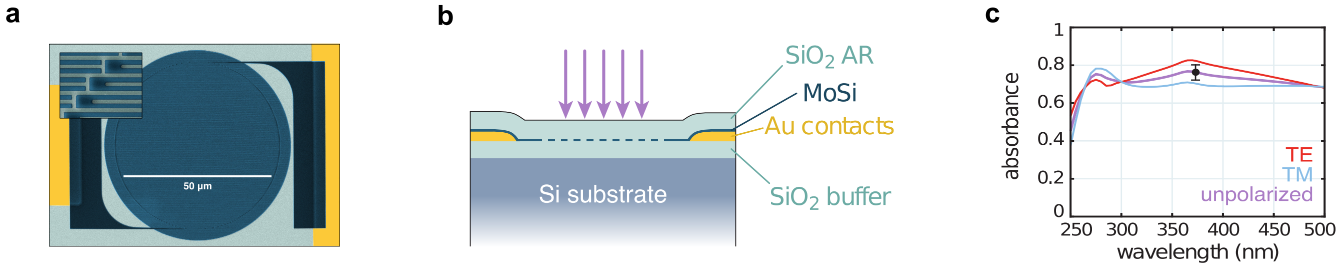

Figure 3. UV superconducting nanowire single photon detector. (a) False-color SEM images of the nanowire pattern. The inset shows a closeup on the nanowire meanders. (b) Optical stack cross-section. (c) Rigorous coupled-wave analysis (RCWA) simulation of absorption by the nanowire layer for TE-polarized (blue), TM-polarized (red), and unpolarized (purple) light. For TE-polarized light, the electric field is oriented parallel to the wires.

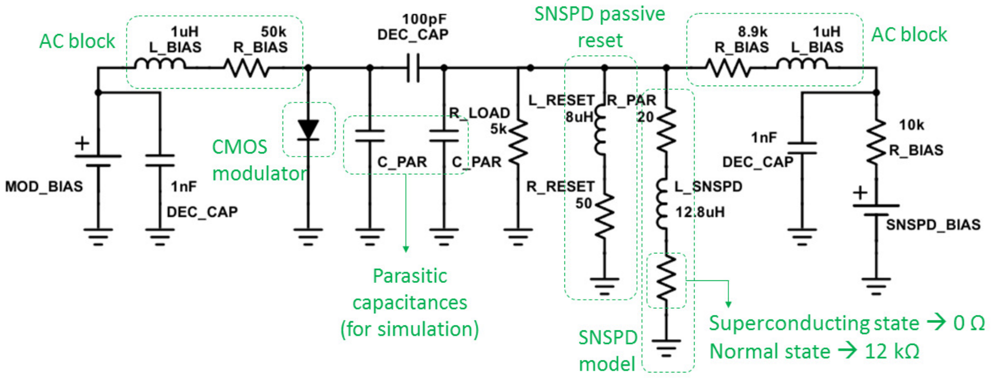

Figure 4. Detailed circuit schematic of the optical readout system. The inductive AC blocks, decoupling capacitors, load resistor and passive reset where implemented in the PCB, to which the CMOS chip and the SNSPD where wirebonded. Due to the modulator differential resistance being around 500 Ω, the 5 kΩ load resistor essentially behaves as an open circuit and does not have any effect in the circuit operation.

Supplementary Discussion 1: Optical readout circuit

Figure 4 shows a schematic of the implemented optical readout circuit. A 100 pF decoupling capacitor is added between the modulator and the SNSPD to allow for different DC bias points but still let the AC signal generated by the SNSPD drive the modulator. Inductive AC blocks are used at the modulator and SNSPD bias inputs to avoid non-DC signals to be coupled back into the bias sources, and 1 nF capacitors to ground are added to filter out high frequency noise in the DC supply.

As described in the main text, when the SNSPD absorbs a photon the developed hotspot resistance diverts the current into the readout, producing a voltage pulse. The SNSPD has a finite hotspot resistance of 12 $k\Omega$, and therefore the current is divided between the non-superconducting SNSPD and the modulator. If the modulator resistance is comparable to the hotspot resistance, current will not leave the nanowire and the hotspot will continue to expand due to Joule heating. Eventually, the SNSPD will "latch" into a normal state[5]. To prevent latching, we used a passive reset circuit ($L_{\text{RESET}} = 8\ \mu$H, $R_{\text{RESET}} = 50\ \Omega$) in parallel with the SNSPD, which provides a low-impedance DC path to ground. This way, we ensure that current will be diverted from the nanowire, allowing for the hotspot to thermally relax and for the SNSPD to return to its superconducting state. The values for L_{RESET} and R_{RESET} were optimized to (1) ensure that any leftover current flowing through the nanowire is eventually sunk into the passive reset branch (with $R_{\text{RESET}} = 50\ \Omega$, 99.6% of the residual current is redirected to the reset) and (2) ensure the time constant of the reset branch is slow enough to allow for a voltage signal to develop at the modulator, but fast enough to not be limiting the speed of the readout.

As shown in Fig.1(e) in the main paper, the input resistance ($r = dV_{mod}/dI_{mod}$) of the modulator is strongly dependent on its bias current. Thus, a situation in which the input resistance of the modulator was too high to be successfully driven by the SNSPD was possible depending on the optimal modulator bias point. To account for this, we added a load resistor ($R_{\text{LOAD}} = 5\ k\Omega$) in parallel with the modulator, which we verified the SNSPD can drive. If the modulator input resistance were higher than R_{LOAD}, the SNSPD bias current would mostly be redirected to the load resistor (instead of the modulator), generating a voltage signal that would drive the modulator. Nonetheless, we measured the input resistance of the modulator for our experimental demonstration to be around 500 Ω, in which case the load resistor is essentially a short circuit and does not affect the operation of the readout.

Circuit simulation

LTSpice was used to simulate the circuit depicted in Fig. 4. A 1 μs transient simulation was performed. The SNSPD resistance model was set to go from 0 Ω in the superconducting state to 12 kΩ in the normal state with a rise time of 1 ns. An SNSPD kinetic inductance of 12.8 μH was obtained from a fit to the response of a standalone SNSPD. The modulator diode model was

obtained from a best fit to the experimental IV curves. The parasitic capacitances C_{PAR} were fit to the experimental readout pulses, resulting in a value of 80 pF at the SNSPD side. At the modulator side, a parasitic capacitance of 70 pF was obtained for a 40 μA bias current, while for a 26 μA bias current it was 40 pF. Different values are expected at different bias points due to the change in the diffusion capacitance of the modulator.

Supplementary Discussion 2: AC electrical power dissipation

As stated in the main text, the AC electrical power dissipation is orders of magnitude lower than its DC counterpart because of the very low voltage amplitudes present in the system. The total AC electrical power dissipation can be written as:

$$P_{AC} = C * V_{AC}^2 * f \tag{1}$$

In the above equation, C corresponds to the input capacitance of the modulator, which is <200 pF as discussed in Supplementary Discussion 1 above. V_{AC} is the peak to peak amplitude of the AC signal, which is <2 mV in our case. Finally, f is the frequency at which readout pulses are generated. For a frequency $f = 1 \times 10^9$ readout pulses per second, the total AC power dissipation is <0.8 μW, two orders of magnitude lower than the DC power, which is 20-40 μW.

Supplementary Methods 2: Data treatment for optical readout pulses

Due to the 30 dB optical coupling losses and the use of Erbium Doped Fiber Amplifiers (EDFAs) both at the input and output of the cryostat, our optical readout demonstration suffered from a low Signal to Noise Ratio (SNR). We applied some digital filtering to the readout waveforms to compensate for this low SNR.

Figure 5 depicts the data treatment process:

- A single optical readout pulse, which as discussed has a low SNR, is shown in green in Fig. 5(a).
- To reduce the noise, we configured the oscilloscope to take an average of 500 pulses, shown in red in Fig. 5 (a).
- As shown in Fig. 5(b), after the averaging a low frequency sinusoidal component at 1 MHz is present. To remove it, we calculated the Fast Fourier Transform (FFT) of the readout signal and substituted the frequency component at 1 MHz by the interpolation of its two nearest neighbors. We also applied a low pass digital filter to remove high frequency noise above 30 MHz. The FFT of the signal before and after the filtering step is shown in Fig. 5 (b). The resulting waveform is shown in black in Fig. 5 (a), and is the waveform reported in the main text.

Supplementary Data 2: Optical readout extended data

In this section we include additional data corresponding to the characterization of the optical readout system.

Figure 6(a) shows a single readout pulse for a modulator bias of 25 μA and an SNSPD bias of 6 μA. As expected, a decrease in the bias current of the modulator results in a smaller amplitude pulse due to a reduced modulation efficiency. A 25 μA bias current corresponds to a modulation efficiency close to 4000 pm/V, which reduces the peak to peak amplitude to around 150 mV (compared to the 200 mV amplitude obtained with 40 μA bias, see Fig. 3(d) in the main text).

Figure 6(b) shows the counts recorded with a pulse counter for different UV powers and different SNSPD bias currents. The modulator bias current was kept at 40 μA and the readout input optical power to the cryostat was 1 mW. Above 6.6 μA bias current the SNSPD undergoes relaxation oscillations[6] and is not photosensitive anymore. As shown in Fig. 6(c), the number of generated pulses depends linearly on the UV optical power hitting the SNSPD. As characterized in[4], the internal efficiency of the SNSPD decreases for decreasing bias currents. This explains why the number of recorded counts in Fig. 6(c) is smaller for lower bias currents.

The solid lines in Fig. 6(c) showing the expected number of counts for each incident laser power are obtained by using the number of counts recorded experimentally for the lowest UV laser power, and assuming a perfectly linear detector, such that:

$$counts_{expected}(P_{in}) = P_{in}\frac{counts_{measured}(P_{min})}{P_{min}} \tag{2}$$

In our case, P_{min}=25 nW.

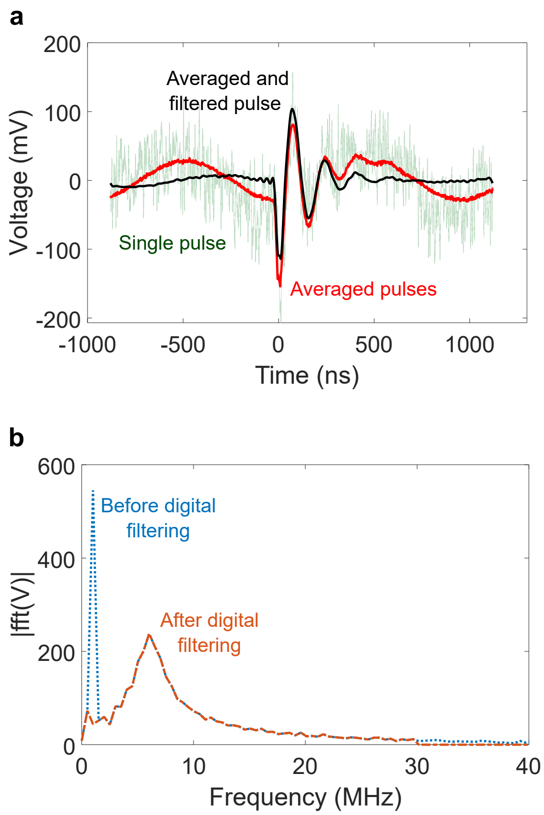

Figure 5. Filtering of optical readout pulses. (a) Optical readout signal in the time domain. Light green shows a single optical readout pulse, red corresponds to an average of 500 pulses, and black shows the averaged and digitally filtered readout. The latter corresponds to the waveform reported in the main text. (b) Magnitude of the FFT of the readout signal before (blue) and after (orange) the digital filtering step. A low pass filter with 30 MHz cutoff is applied, and the frequency component at 1 MHz is interpolated using the nearest neighbors. The shown pulse corresponds to a modulator bias current of 40 μA and and SNSPD bias of 6 μA.

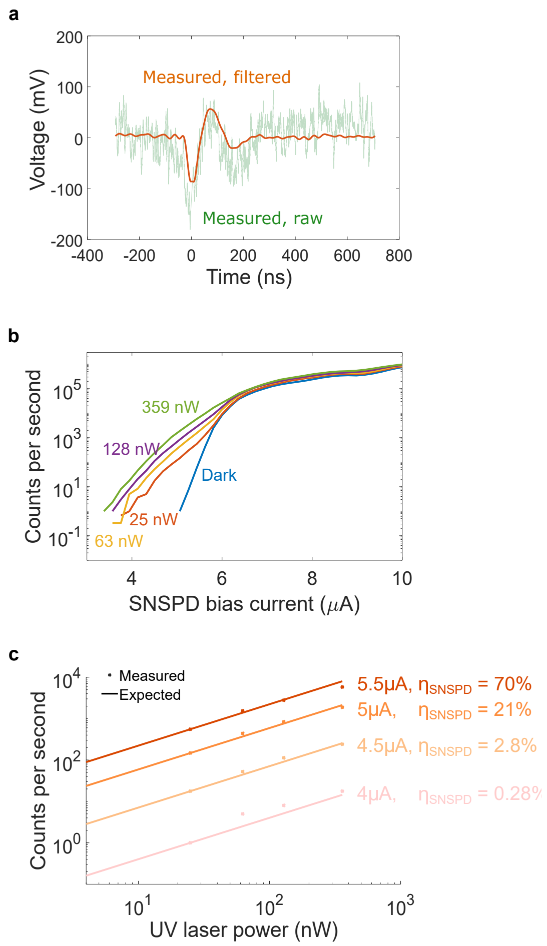

Figure 6. Optical readout additional data. (a) Readout pulse for a modulator bias current of 25 μA and an SNSPD bias of 6 μA. Light green corresponds to a single pulse, and orange shows an averaged and filtered pulse. (b) Counts per second as a function of SNSPD bias and UV power hitting the SNSPD. Above 6.6 μA the SNSPD undergoes relaxation oscillations. (c) Counts per second as a function of the UV optical power hitting the SNSPD for 4 different bias currents. Dots show measured values, and lines show the expected value assuming a linear detector. Less counts are measured for lower bias currents due to a decrease in the SNSPD internal efficiency.

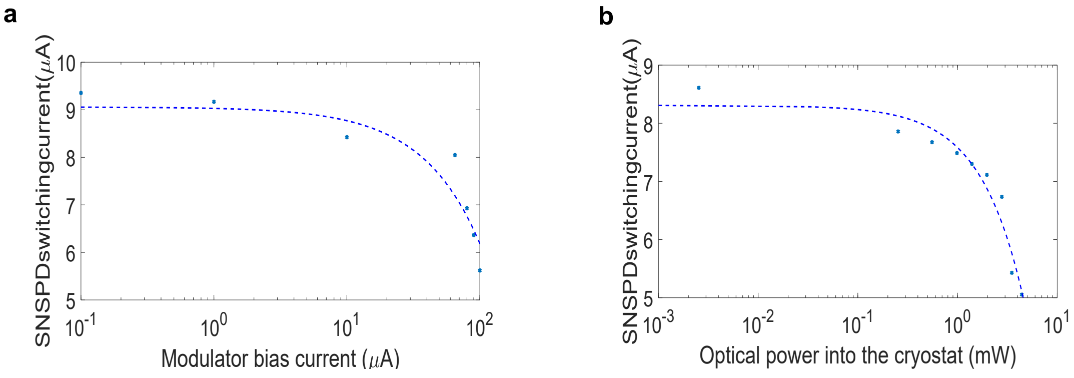

Figure 7. SNSPD switching current as a function of the modulator bias current (a) and as a function of the input optical power into the cryostat (b). Dots are measured values, and dashed lines are a linear fit as a guide to the eye. Modulator bias currents up to 70 μA and optical powers up to 2 mW result in switching currents above 7 μA. The switching current without any extra heat load is about 9 μA.

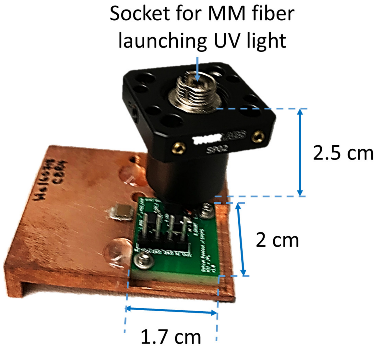

Figure 8. Picture of the optical readout system. A lens tube is used to launch UV light into the SNSPD. The lens tube is used to isolate the SNSPD from scattered 1550 nm light used for the optical readout.

Supplementary Data 3: Optical readout heat load

One of the main concerns of operating the CMOS modulator at the same temperature stage as the SNSPD is the possibility of thermal crosstalk between the two devices. It is very likely that the modulator is locally at a temperature higher than that of the cryogenic environment (which is around 3.6 K) for two reasons: (1) Ohmic heating resulting from the forward bias operation of the modulator and (2) free carrier absorption. Additionally, high input optical powers could also result in an increase in the temperature of the cryogenic environment. Excessive heating could be fatal for the operation of the SNSPD since its switching current depends strongly on its temperature.

We recorded the SNSPD switching current for different modulator bias points and different input optical powers to the cryostat. The results are shown in Fig. 7. Increasing the modulator bias current or the optical power results in a decrease in the SNSPD switching current caused by an increase in its temperature. This has a direct effect in the quality of the optical readout: a lower switching current results in a lower driving signal at the modulator, which translates into a lower wavelength shift and shallower modulation.

If we set the boundary of acceptable heating to a 20% decrease in the switching current (from 9 μA without any heat load to 7 μA), modulator bias currents up to 70 μA and input optical powers up to 2 mW are acceptable. The operating conditions for the experimental results presented in this work are 25 - 40 μA of modulator bias current and 1 mW of input optical power, which are within the acceptable bounds. Based on measurements of the switching current as a function of temperature on a standalone SNSPD, switching currents above 7 μA are obtained for temperatures $<$3.8 K. This means that our forward biased CMOS modulator results in a minimal increase in the SNSPD temperature of $<$200 mK. Moreover, and as discussed in Supplementary Discussion 4, simple improvements to the optical coupling to the CMOS chip could decrease the heat load by 2 orders of magnitude, which would eliminate any detrimental effects to the SNSPD operation caused by the optical readout.

Supplementary Discussion 3: Pulse counting

SNSPD and UV laser misalignment

We can estimate the number of counts per second we would expect from the number of photons hitting the SNSPD:

$$cps = \eta_{snspd} * \eta_{misalignment} * \frac{P_{int}(r_{snspd})}{P_{int}(r \to \infty)} * \phi_{TOT} \tag{3}$$

ϕ_{TOT} is the total flux of photons, which for a power of 360 nW (the power used for the waveforms shown in figure 3(c,d) of the main paper) and a wavelength of 373 nm is 7×10^{11} photons per second. $\eta_{snspd} = 0.7$ and $r_{snspd} = 28$ μm are the SNSPD internal efficiency and radius, respectively.

$P_{int}(r)$ is the power contained in a circular aperture of radius r by a gaussian beam centered in the aperture, which is given by:

$$P_{int}(r) = P_{TOT} * (1 - e^{\frac{-2r^2}{w^2}}) \tag{4}$$

P_{TOT} is the total power of the gaussian beam, and w is the beam waist radius, which can be approximated as:

$$w(d) = NA * d \tag{5}$$

NA is the numerical aperture of the multimode fiber used to launch the UV light and d is the distance between the tip of the fiber and the SNSPD surface. In our case, NA=0.22 and $d \approx 2.5$ cm (see Fig. 8).

$\eta_{misalignment}$ accounts for the misalignment between the center of the SNSPD and the center of the UV gaussian beam, which decreases the number of photons incident on the SNSPD. It is easy to show that $\eta_{misalignment}$ is given by:

$$\eta_{misalignment} = \frac{e^{\frac{-2r_0^2}{w^2}} \int_0^{r_{snspd}} \int_0^{2\pi} r\, exp(-2r^2/w^2) exp(4\, r_0\, r\, cos(\theta)/w^2)\, d\theta\, dr}{(\pi/2) w^2 (1 - e^{\frac{-2r^2}{w^2}})} \tag{6}$$

r_0 is the distance between the center of the gaussian beam and the center of the SNSPD.

Figure 9 plots Eq. 3 as a function of the misalignment between the UV beam and the SNSPD for the parameters corresponding to our experimental demonstration. If the SNSPD and the UV fiber were perfectly aligned, we would expect around 2.5×10^7 cps, or one count every 50 ns. It is clear by observing Fig. 3(c) in the main paper that this is not the case for us. This is expected, since our assembly did not have a mechanism to align the UV beam to the center of the SNSPD (the holes for attaching the lens tube holding the UV fiber were drilled before positioning the SNSPD).

We can use the time traces recorded with the oscilloscope to estimate the number of photons impinging on the SNSPD, in which case we get about 10 counts every 60 μs, or about 2×10^5 cps (see Fig. 3(c) in the main text). If we assume this is the number of photons hitting the SNSPD, we conclude that the UV laser beam and the SNSPD were misaligned by approximately 8 mm, which is plausible given our coarse relative positioning between the SNSPD and the lens tube.

Pulse counting efficiency

Since we have estimated the number of photons incident on the SNSPD, we can calculate the pulse counting efficiency we obtained for the voltage threshold we set for the pulse counter. For a bias current of 5 μA we obtained around 2×10^3 cps (see Fig. 3(e) in the main paper), which translates into an efficiency $\eta \approx 1\%$ for 2×10^5 photons per second incident on the SNSPD.

This efficiency is mainly limited by the low SNR of our optical readout signal due to the 30 dB loss in the input-output optical coupling to the CMOS chip, caused mainly by the use of non-optimized grating couplers. These losses directly result in a 30 dB hit in the signal to noise ratio, which is further deteriorated by the use of EDFAs with a high noise figure of about 6 dB. With a low SNR signal, we needed to set a higher voltage threshold for the pulse counter in order to avoid noise events to be counted as readout pulses, which causes certain fraction of real pulses (pulses corresponding to a photon detection event) to be missed (see Supplementary Discussion 4) and thus decreases the pulse counting efficiency.

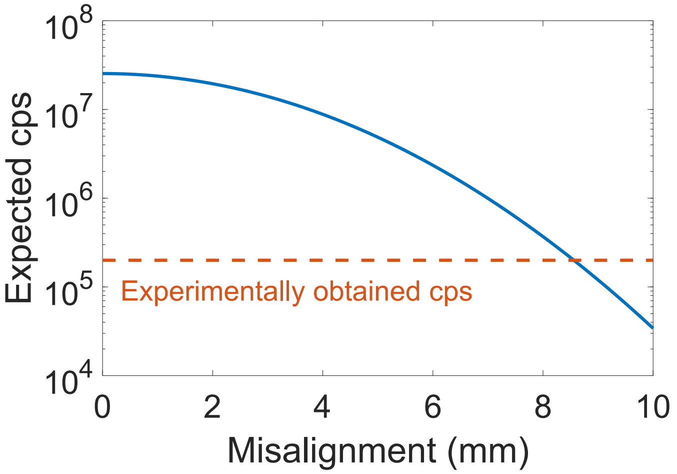

Figure 9. Blue: Expected number of counts as a function of the misalignment between the center of the UV beam and the center of the SNSPD for an input UV power of 360 nW. Orange: inferred cps from the recorded oscilloscope traces.

Supplementary Discussion 4: Potential for improved performance

Simple improvements in the optical coupling to the chip would allow for a substantial decrease in the optical power that was needed for this demonstration. As has been mentioned previously, the readout is limited by a low SNR, which is mainly due to the 30 dB insertion loss introduced by the optical coupling in and out of the CMOS chip.

These high losses are not intrinsic to the technology: grating couplers with $> 90\%$ efficiency have been demonstrated in zero change CMOS[7]. Thus, we believe that by the use of optimized grating couplers and a better polish angle control, insertion losses could be reduced to about 3-5 dB after cooling down to cryogenic temperatures.

Reducing the optical loss by 25 dB would allow us to obtain the same output signal with 25 dB lower input optical power, from 1 mW to about 5 μW. This would impact the quality of the readout in 2 different ways. First, it would decrease the heat load to the cryostat, reducing the operating temperature of the SNSPD and thus increasing its switching current (from ≈ 7.5 μA to ≈ 9 μA) as observed in Fig. 7, resulting in a 20% increase in the electrical signal driving the modulator. Assuming a linear dependency between electrical signal and modulation depth, which is a good approximation given the small amplitude signals that develop in our system, the generated readout signal would increase by 20%. Second, a decrease in the optical coupling loss would eliminate the need for an input EDFA, which would increase the SNR of the readout signal by a factor equal to the Noise Figure of the amplifier, which in our case is specified to be > 6 dB.

Thus, the improvement in the optical coupling loss would result in a ≈ 7 dB increase in the SNR of the readout signal, which would allow for a much higher pulse counting efficiency of our optical readout. Because of the low SNR, our demonstration was mainly limited by the need to set the threshold for the pulse counter at a level far enough from the noise floor so as not to get any false count from noise events. As a consequence, a great part of the pulses corresponding to detected photons were actually not counted because they didn't overcome the pulse counter threshold.

Using measured data, we fitted the noise to a gaussian distribution to estimate its variance and obtained the signal power by integrating a single readout pulse, which resulted in an SNR=1.83. Figure 10 shows the readout waveform we would obtain if there was no noise (orange), for the experimental SNR=1.83 (green) and for the SNR=10 we would obtain with improved optical coupling (purple). Clearly, an increase in the SNR would allow for a lower threshold for the pulse counter, which would increase the number of pulses detected and would result in a detection efficiency approaching that of the SNSPD, which is close to 70% for our detector.

Input impedance

As discussed in the main paper, superconducting devices are not capable of driving high input impedance loads. For instance, SNSPDs typically drive an amplifier with a 50 Ω input impedance, but our modulator showed and input impedance of around 500 Ω. As a consequence, we added a passive reset branch to sink all the current still flowing through the SNSPD when in its normal state because of the impedance mismatch, which allowed it to recover to the superconducting state at the expense of increasing the complexity of the system and adding parasitics that reduce the speed of the optical readout system.

Nevertheless, the differential resistance of an ideal diode is given by $r_d = kT/qI$, which for a 40 μA current at 4K gives $r_d = 10\ \ \Omega$. Clearly, the modulator resistance in our demonstration is limited by its series resistance, which is mainly due to the

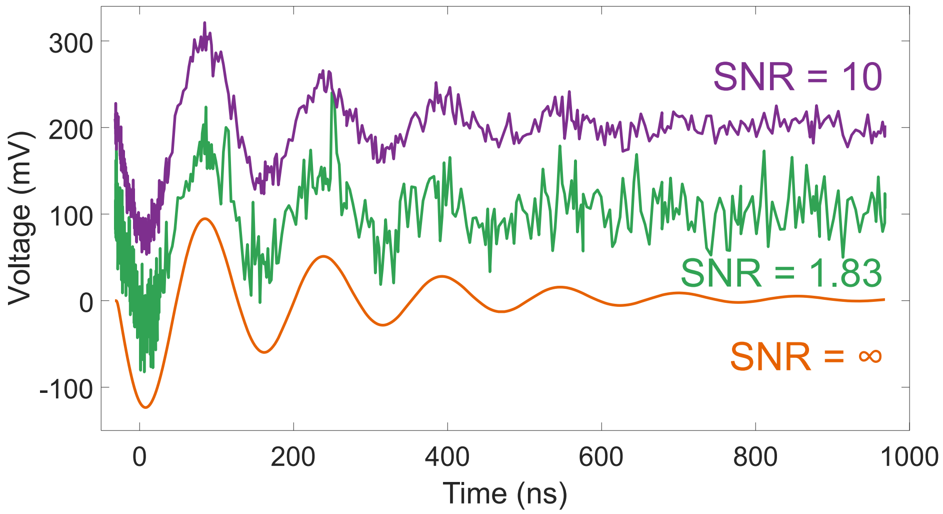

Figure 10. Readout waveforms for different SNR. Orange corresponds to SNR = ∞, green to the experimentally obtained SNR of 1.83, and purple to the attainable SNR of 10 with improved optical coupling. Each waveform is offseted 100 mV for ease of visualization.

resistance of the quasi-neutral regions of the pn junctions in the ring (the estimated resistance due to the metal layers used for signal routing is only $R = 12\ \Omega$). Several techniques exist to reduce the series resistance in our device. For instance, we could reduce the width of the intrinsic regions in the T-junction design, or we could increase the doping of the p and n regions to compensate for the partial ionization at low operating temperatures.

Notice that reducing the series resistance would not have any detrimental effect in the modulation depth, since the voltage drop through the pn junction (which is the voltage that modulates the output signal) is still $V_{pn} \sim I_{SNSPD} \times r_d$, which is independent of the series resistance. On the other hand, reducing the series resistance would allow the modulator to present a much lower input impedance to the superconducting device, considerably reducing the impedance mismatch and making the use of the passive reset branch unnecessary.

Thus, we can conclude that by optimizing the modulator to have negligible series resistance at cryogenic temperatures, our modulator could have shown an input impedance of about 10 Ω (∼ 20-30 Ω if accounting for the resistance of the metal routing lines) for the 40 μA bias current we demonstrated experimentally, without incurring in any reduction in the output signal. This would have allowed us to eliminate the passive reset branch, reducing both parasitic effects and the system footprint. Furthermore, having a modulator with such low input impedance would allow for direct readout of superconducting devices with even lower impedance than SNSPDs, such as SFQ circuits.

Extension to IR SNSPDs

As already discussed, in this work we used a UV SNSPD, which is advantageous because of its large hotspot resistance, large switching current, high critical temperature and insensitivity to 1550 nm light, which avoids scattered readout light to generate counts on the SNSPD.

The extension of our readout architecture to infrared (IR) SNSPDs is of great interest, since these are more widely deployed and have a larger application space. Because the operating principle of an SNSPD is the same regardless of its operating wavelength, the readout architecture presented in this work can be extended to any SNSPD. Nevertheless, additional constraints arise when operating with an IR SNSPD:

1. Scattered readout light could generate counts on the SNSPD. Several approaches exist to minimize this crosstalk. For instance, the use of a lens tube (or some other physical barrier) to isolate the SNSPD from the readout light (as shown in Fig. 8) should prevent most of the readout photons from impinging on the SNSPD. Additionally, and as discussed above, reducing optical coupling losses would allow for a close to three orders of magnitude reduction in the necessary input power for readout, further decreasing the number of scattered photons.

2. Typical IR SNSPDs have a lower switching current (≈ 4 μA) and a lower hotspot resistance (≈ 1 $k\Omega$) than UV SNSPDs, which would decrease the generated driving signal at the modulator to less than 1 mV. This could be compensated by an increase in the SNR of the readout signal (as we have discussed previously), which would decrease the necessary

optical transmission change for detection of a pulse. Additionally, the modulator bias current could be increased so that a higher modulation efficiency is achieved, or the sensitivity of the receiver chain detecting the readout light increased (for example, by using a coherent detection scheme).

3. Typical IR SNSPDs have a lower critical temperature than UV SNSPDs, and are usually operated at < 1 K, where the available cooling power is highly constrained. We believe our forward biased optical modulators could operate at a 1 K temperature due to the ionization generated by the small DC current flowing, which decreases the impact of carrier freezeout. Nevertheless, additional work is necessary to ensure that the modulator power consumption is low enough to be compliant with the available cooling power at this temperature stage. Alternatively, the readout modulator could be operated at the 4 K stage, and connected to the 1 K stage through a high speed microwave cable, which would present a minimal heat load due to the fact that the temperature difference between the two stages is very small.

Summarizing, the same operating principle presented in this work can be applied to the readout of IR SNSPDs with minor improvements to the system.

References

1. Gehl, M. *et al.* Operation of high-speed silicon photonic micro-disk modulators at cryogenic temperatures. *Optica* **4**, 374–382 (2017).

2. Nguyen, H. T., Baker-Finch, S. C. & Macdonald, D. Temperature dependence of the radiative recombination coefficient in crystalline silicon from spectral photoluminescence. *Appl. Phys. Lett.* **104**, 112105, DOI: 10.1063/1.4869295 (2014).

3. Ichimura, M., Tajiri, H., Ito, T. & Arai, E. Temperature dependence of carrier recombination lifetime in si wafers. *J. The Electrochem. Soc.* **145**, 3265–3271, DOI: 10.1149/1.1838796 (1998). http://jes.ecsdl.org/content/145/9/3265.full.pdf+html.

4. Wollman, E. E. *et al.* UV superconducting nanowire single-photon detectors with high efficiency, low noise, and 4 K operating temperature. *Opt. Express* **25**, 26792–26801 (2017).

5. Kerman, A. J., Yang, J. K. W., Molnar, R. J., Dauler, E. A. & Berggren, K. K. Electrothermal feedback in superconducting nanowire single-photon detectors. *Phys. Rev. B* **79**, 100509, DOI: 10.1103/PhysRevB.79.100509 (2009).

6. Hadfield, R. H., Miller, A. J., Nam, S. W., Kautz, R. L. & Schwall, R. E. Low-frequency phase locking in high-inductance superconducting nanowires. *Appl. Phys. Lett.* **87**, 203505, DOI: 10.1063/1.2130525 (2005).

7. Notaros, J. *et al.* Ultra-efficient CMOS fiber-to-chip grating couplers. In *Optical Fiber Communication Conference*, DOI: 10.1364/OFC.2016.M2I.5 (2016).
